# Prevalence of Malaria Parasite among Pregnant Women Attending to Saudi Kassala Teaching Hospital in Kassala State, Eastern Sudan

**DOI:** 10.1155/2023/2289552

**Published:** 2023-11-30

**Authors:** Alnaeem Abdalla Idris Nour, Tafawl Ibrahim Karrar, Mohamed Hassan Ahmed Kebayer, Nada Ali Abd Elwahid Mohamed, Khadega Suleiman Mohammed Zarroug, Hajrhma Ismael Hajrhma Mohammedahmed, Mohamed Ahmed Salah Mohamed Ahmed

**Affiliations:** ^1^Department of Parasitology and Medical Entomology, Medical Laboratory Sciences Program, Faculty of Medicine and Health Sciences, University of Kassala, Kassala, Sudan; ^2^Department of Microbiology and Parasitology, Faculty of Medicine and Health Sciences, University of Kassala, Kassala, Sudan; ^3^Department of Community Medicine, Faculty of Medicine and Health Sciences, University of Kassala, Kassala, Sudan

## Abstract

**Objective:**

Malaria during pregnancy is a priority area for malaria research and control as pregnant women represent a high risk group for severe malaria, and the presentation of malaria during pregnancy varies according to the level of transmission in the area; so the aim of this study is to determine the prevalence rates of malaria parasite among pregnant women attending to Saudi Kassala Teaching hospital in Kassala state, 2022.

**Methods:**

A cross-sectional study was carried out in Saudi Kassala Teaching hospital in Kassala State. This study involved one hundred and eighty-five blood samples collected from pregnant women who was then examined by using blood films and ICT for malaria, and the data were collected by a structured questionnaire and analyzed using SPSS version 21.

**Results:**

The prevalence of malaria among pregnant women was 2.2% (95% CI: 0.006–0.054). There was no significant difference among the different age groups with respect to the prevalence of malaria (*P* value = 0.483). The prevalence of malaria in rural residency was 2.2%, and this was significantly more common than the urban residency (*P* value = 0.021). When compared across the gestational trimesters, there was no significant difference between them (*P* value = 0.518). The number of gravidity is not related to malaria infection (*P* value = 0.737). The presence of symptom compliant of malaria during pregnancy does not suggest the presence of malaria (*P* value = 0.152). No difference was found between the different educational levels with respect to the prevalence of malaria (*P* value = 0.362). The result showed that there was 1 (0.5%) negative result in ICT which was positive in blood film for malaria (BFFM) and there were 3 (1.6%) positive malaria parasites by both methods in all 185 samples with statistically insignificant differences (*P* = 0.703).

**Conclusion:**

*Plasmodium falciparum* was only species detected in this study. Malaria among pregnant women was more prevalent in rural areas. However, other factors such as age, gestational age, gravidity, and educational level do not affect the prevalence of malaria in pregnant women. The presence of symptomatic compliant of malaria during pregnancy does not suggest the presence of malaria. The use of ICT or BFFM has similar diagnostic outcome for malaria in pregnancy.

## 1. Introduction

Malaria is caused by *Plasmodium* species, and *P. falciparum* is more common in Africa. According to WHO, Sudan carries the heaviest burden accounting for 61% of deaths in the Eastern Mediterranean [1]. There are geographically variations in the epidemiology of this [2]. Of all the species of *Plasmodium* parasites, *Plasmodium falciparum* is responsible for the highest disease burden [3]. The other species have low disease burdens [4]. An interaction between the host, the vector, and the parasite is needed for propagation of the disease [5]. Other rare modes of transmission include transplacental, through blood transfusions, or through contaminated needles or syringes [6]. The early symptoms of malaria are flu-like presentation which is similar among the different species of malaria [7]. The main symptom of malaria is fever, and other symptoms may include nausea, vomiting, anorexia chills, headache, and myalgia [8]. In pregnancy, malaria is a major health issue; it has been associated with many maternal and fetal complications such as anemia with pregnancy and stillbirth, respectively [9]. In sub-Saharan Africa, malaria in pregnancy is a disease burden caring mortalities as well as morbidities [10]. This is mainly due to *Plasmodium falciparum* [11]. In areas with low malarial transmission, 25% of severe anemia with pregnancy is due to malaria, and placental malaria doubles the risk of low birth weight. However, in areas with high malarial transmission, malaria-related fetal growth restriction may be twice as high as malaria-related preterm delivery [12]. Pregnant women are more liable to malaria which adversely affects pregnancy. It is one of the chief causes of maternal mortality in Sudan, especially with *Plasmodium falciparum* which cause high levels of parasitemia, hypoglycemia, acute pulmonary edema, fetal distress, premature labor, spontaneous abortions, and still births [13]. The present study aims to determine the prevalence rate of malaria parasite among pregnant women attending to Saudi Kassala Teaching hospital in Kassala State, as the malaria trend increases by as much as over 40% between 2015 and 2020 [1].

## 2. Materials and Methods

### 2.1. Study Design and Study Population

The study was a descriptive cross-sectional hospital-based study, conducted from February to September 2022. It was conducted in Saudi Kassala Teaching hospital in Kassala state among 185 pregnant women attending the hospital for either monthly follow-up visit or delivery. Kassala state is located in the east of the Sudan. It has a total population of 1.5 million [14].

This state is served by Saudi Kassala teaching hospital, which is the only hospital that provides antenatal and delivery service to the state population. In 2022, the hospital had 7498 spontaneous vaginal deliveries, 18 assisted vaginal deliveries through ventouse suction, and 5,586 cesarean section [15].

Sample size was calculated using the following formula:

(1) $$\begin{array}{c} n=\frac{z^{2}\text{pq}}{d^{2}}, \end{array}$$

where *z* is the normal standard deviation (1.96), *p* is the frequency of occurrence of an event, *q* is the frequency of nonoccurrence of an event, and *d* is the degree of precision (0.05) [16].

So, when the margin of error is 5%, confidence level is 95%, and prevalence is 13.7% [17].

(2) $$\begin{array}{c} n=\frac{1.96*1.96*0.137*\left(1-0.137\right)}{0.05*0.05}\\ =182\text{ samples}. \end{array}$$

The calculated sample is 182; however, a sample of 185 was taken for more accuracy and precision.

#### 2.1.1. Inclusion Criteria

Inclusion criteria included pregnant women attending Saudi Kassala Teaching hospital from February to September 2022.

#### 2.1.2. Exclusion Criteria

Exclusion criteria included pregnant women referred from outside of the state of Kassala and pregnant women who were diagnosed with malaria by laboratories other than the Saudi Kassala Teaching hospital laboratory.

### 2.2. Methodology of Laboratory Investigations

A 5 ml of blood sample was collected from pregnant women in EDTA container. Thin and thick microscopic examination and immunochromatographic test (ICT) were used for confirming the diagnosis.

#### 2.2.1. Blood Films

Thick and thin blood films were air-dried. Methanol was used to fix the thin films. 10% Giemsa solution was used to stain the films, and the stain was allowed for 10 minutes, then washed with clean water, and left to air-dry. 100x magnification lenses under oil immersion were used to view the films [18].

#### 2.2.2. Rapid MAT Test

This test has a membrane strip coated with monoclonal antibodies. The first test line for *Plasmodium falciparum* is coated with monoclonal antibodies against histidine-rich protein 2 while the second line for *Plasmodium vivax* is coated with monoclonal antibodies specific to lactate dehydrogenase [19].

At room temperature, port A in the test kit was filled with 5 *µ*l of anticoagulated blood using a micropipette, and two drops of buffer was added in port B using plastic dropper. The results were interpreted after 20 minutes [18].

### 2.3. Data Collection and Analysis

After estimating the sample, a questionnaire was designed to collect the demographic data as well as data related to the gestational age, gravidity, and malaria symptoms during pregnancy.

The data were analyzed using frequencies and cross-tabulation using chi-square in Statistical Package for Social Science (SPSS) computer program version 21.

### 2.4. Ethical Approval and Consent to Participate

All the examination protocols were approved by the ethical committees of both the faculty of medicine and health sciences at the University of Kassala and Saudi Kassala Teaching hospital in Kassala State. The informed written consent was taken from each pregnant women participated in this study.

Pregnant women who are less than 16 years of age were married, and as a result, they were considered emancipated, and the informed written consent was taken accordingly.

Informed written consent from illiterate pregnant women was taken with the participation of the husband as a legal guardian.

Of the 185 women approached, the data were successfully collected and no refusal was noted.

All the methods were carried out in accordance with local regulations and guidelines.

## 3. Results

Of the total 185 pregnant women selected, the response rate was 100%. The result showed that out of 185 pregnant women, only 4 (2.2%, 95% CI: 0.6–5.4) were infected with malaria parasite, and all of them were infected by *Plasmodium falciparum* (Table 1).

The number of samples examined for malaria parasites among pregnant women in each age group was 85, 88, and 12 in age groups of 15–25 years, 26–35 years, and 36–45 years, respectively; the result showed that 3 (3.5%) were positive in the age group 15–25 years and 1 (1.1%) in the age group of 26–35 years. There was no significant difference among the different age groups with respect to the prevalence of malaria at *P* value = 0.483 (Table 2).

The result revealed that all the positive cases of malaria were found among pregnant women living in rural residency 4 (5.0%) compared with 0 (0%) positive cases in the urban residency, and these differences in rates were significant at *P* = 0.021 (Table 3).

The result showed that out of the 70 illiterate pregnant women, 3 (3.4%) were positive for malaria parasite, and out of the 39 who had secondary educational level, 1 (2.6%) was positive for malaria parasite, while the other educational groups level was negative for malaria parasite with statistically insignificant differences in rates at *P* = 0.362 (Table 4).

When compared across the gestational trimesters, the result showed that pregnant women in first and second trimesters were negative for malaria, while, in third trimester, the result showed 4 (2.8%) positive for malaria, with statistically insignificant differences rate at *P* = 0.518 (Table 5).

The result also showed that the number of gravidity is not related to malaria infection because there were 1 (2.3%), 1 (3.0%), 2 (3.0%), and 0 (0.0%) positive case among primigravidity, second gravidity, multigravidity, and grand multigravidity, respectively, with statistically insignificant differences in rate at *P* = 0.737 (Table 6).

Out of the 39 pregnant women complaining of malaria symptoms which include fever, nausea, vomiting, anorexia, chills, headache, and myalgia [8], only 2 (5.1%) were positive for malaria. Among the remaining 146 pregnant women who had no symptoms, only 2 (1.4%) were positive for malaria parasite; thus, the presence of malaria symptoms does not suggest the presence or absence of malaria (Table 7).

The result showed that there was 1 (0.5%) negative result in ICT which was positive in BFFM and there were 3 (1.6%) positive malaria parasites by both methods in all 185 samples, with statistically insignificant differences at *P* = 0.703 (Table 8).

## 4. Discussion

The study showed that the prevalence of malaria among pregnant women was 2.2% (95% CI: 0.6–5.4). Meanwhile, the department of statistics at Saudi Kassala Teaching hospital recorded a prevalence of 1.79% in 2020 and 1.57% in 2011. This is relatively similar to the results of our study, and these indicate that malaria among pregnant women was relatively stable throughout the last 3 years [15].

According to our study, *Plasmodium falciparum* was the only species of malaria parasite detected, and this was similar to that of a study done in Al Jabalian Locality, White Nile state, Sudan, which has similar result [20]. This also is in accordance with the World Health Organization (WHO), where *Plasmodium falciparum* is the predominate species in Africa [21].

We found that the highest prevalence of malaria in pregnancy was 3.5% recorded among age group of 15–25 years. However, there was no significant association between the prevalence of malaria and the age group of pregnant women, and this finding is similar to another study done in eastern Sudan [17]. This similarity may be because both studies were conducted in same region, i.e., eastern Sudan.

Malarial infection was significantly higher in women from rural areas compared to woman from urban areas; this finding was similar to the findings reported by Omer and colleagues [10]. This higher prevalence among rural areas may be attributed to the low socioeconomic status at the rural communities.

Even though malaria prevalence is relatively higher in the noneducated group (4.3%), there is no significant association between the education level and malaria infection, and this finding agrees with the finding reported by Suliman and colleagues [20].

There is no association between the gestational age and malaria, and this finding is similar to that of a study done by Tahita and colleagues [22].

This study showed no significant association between malaria and gravidity, and this finding was similar to that of another study done in eastern Sudan by Adam and colleagues [17].

Our study shows that symptoms of malaria were not discriminative for malaria infections (value = 0.152). This is similar to the findings of the study done by Tahita and colleagues [22]. The difficulty of discrimination may be because certain malaria-related symptoms may mimic pregnancy-related symptoms.

When the blood films and ICTs were compared for the diagnosis of malaria parasite, the difference between the two modalities was not statistically significant (*P* value = 0.751). This was similar to the results obtained by Latha and colleagues where rapid diagnostic test was found equally sensitive to microscopy [23].

## 5. Conclusions

*Plasmodium falciparum* was the only species detected in this study.

Malaria among pregnant women was more prevalent in rural areas. However, other factors such as age, gestational age, gravidity, and educational level do not affect the prevalence of malaria in pregnant women.

The presence of symptomatic compliant of malaria during pregnancy does not suggest the presence of malaria.

The use of ICT or BFFM has similar diagnostic outcome for malaria in pregnancy.

## Figures and Tables

**Table 1:** Overall prevalence of malaria among pregnant women.

Total no.	Number of positive (*P. falciparum*)	Number of negative	Percentage (%)	95% confidence interval	
				Lower	Upper
185	4	181	2.2	0.6	5.4

**Table 2:** Prevalence of malaria among pregnant women by age group.

Age group (years)	Number examined	Number of positive	Percentage	95% confidence interval	
				Lower	Upper
15–25	85	3	3 (3.5%)	0.7	10
26–35	88	1	1 (1.1%)	0.0	6.2
36–45	12	0	0 (0.0%)	0.0	26.5
Total	185	4	4 (2.2%)		

Chi-square *P* value = 0.483.

**Table 3:** Prevalence of malaria among pregnant women by residency.

Resident	Number examined	Number of positive	Percentage	95% confidence interval	
				Lower	Upper
Urban	105	0	0 (0.0%)	0.0	3.5
Rural	80	4	4 (5.0%)	1.4	12.3
Total	185	4	4 (2.2%)		

Chi-square *P* value = 0.021.

**Table 4:** Prevalence of malaria among pregnant women by educational level.

Educational level	Number examined	Number of positive	Percentage	95% confidence interval	
				Lower	Upper
Illiterate	70	3	3 (4.3%)	0.9	12.0
Primary	45	0	0 (0.0%)	0.0	7.9
Secondary	39	1	1 (2.6%)	1.0	13.5
University	31	0	0 (0.0%)	0.0	11.2
Total	185	4	4 (2.2%)		

Chi-square *P* value = 0.362.

**Table 5:** Prevalence of malaria among pregnant women by gestational age.

Gestational age	Number examined	Number of positive	Percentage	95% confidence interval	
				Lower	Upper
First	15	0	0 (0%)	0.0	21.8
Second	30	0	0 (0%)	0.0	11.6
Third	140	4	4 (2.8%)	0.8	7.2
Total	185	4	4 (2.2%)		

Chi-square *P* value = 0.518.

**Table 6:** Prevalence of malaria among pregnant women by gravidity.

Gravidity	Number examined	Number of positive	Percentage	95% confidence interval	
				Lower	Upper
Prime	43	1	1 (2.3%)	0.1	12.3
Second	33	1	1 (3.0%)	0.1	15.8
Multi (3 to 4 times)	67	2	2 (3.0%)	0.4	10.4
Grand multi (5 or more times)	42	0	0 (0.0%)	0.0	8.4
Total	185	4	4 (2.2%)		

Chi-square *P* value = 0.737.

**Table 7:** Prevalence of malaria among pregnant women by symptoms.

Symptoms	Number examined	Number of positive	Percentage	95% confidence interval	
Yes	39	2	2 (5.1%)	0.6	17.3
No	144	2	2 (1.4%)	0.2	4.9
Total	185	4	4 (2.2%)		

Chi-square *P* value = 0.152.

**Table 8:** Comparison between BFFM and ICT in diagnosis of malaria among pregnant women.

		BFFM		
		Number of positive	Number of negative	Total
Immunochromatographic test (ICT)	Number of positive	3 (1.62%)	0 (0.0)	3 (1.62%)
	Number of negative	1 (0.54%)	181 (97.84%)	182 (98.38)
	Total	4 (2.16%)	181 (97.84)	185 (100%)

Chi-square *P* value = 0.703.

## Data Availability

The data supporting the current study are available from the corresponding author upon request.
